# Effect of High-Frequency Repetitive Transcranial Magnetic Stimulation on Visual Selective Attention in Male Patients With Alcohol Use Disorder After the Acute Withdrawal

**DOI:** 10.3389/fpsyt.2022.869014

**Published:** 2022-04-29

**Authors:** Zuxing Feng, Qiao Wu, Li Wu, Tingting Zeng, Jing Yuan, Xin Wang, Chuanyuan Kang, Jianzhong Yang

**Affiliations:** ^1^Department of Psychiatry, The Second Affiliated Hospital of Kunming Medical University, Kunming, China; ^2^Department of Substance use Disorders, the Psychiatry Hospital of Yunnan, Kunming, China; ^3^Department of Psychosomatic Medicine, Tongji University School of Medicine, Shanghai East Hospital, Shanghai, China

**Keywords:** alcohol use disorder, cognitive dysfunction, transcranial magnetic stimulation, oddball paradigm, attention bias

## Abstract

**Objective:**

To investigate the effect of high-frequency repetitive transcranial magnetic stimulation (rTMS) on attention cue reactivity in male patients with alcohol use disorder (AUD) after acute withdrawal.

**Methods:**

A total of 90 male patients with AUD who were hospitalized were enrolled and divided into study and waiting groups by a random number table. During the study, 18 patients dropped out. After the alcohol withdrawal symptoms were eliminated, the study group received high-frequency rTMS at 10 Hz for 14 consecutive days, and the waiting group was administrated by sham rTMS. All subjects were evaluated for attention cue reactivity, impulsiveness, cognitive function by oddball paradigm, Barratt Impulsiveness Scale version II (BIS-II), and the Montreal Cognitive Assessment (MoCA) at baseline and after true or sham rTMS.

**Results:**

1. There was no significant difference between the study and the waiting groups regarding the drinking level, cognition level, and demographic data at baseline. 2. In the oddball paradigm, both for alcohol-related and non-alcohol-related cues, the response times were significantly shorter in the study group after rTMS treatment than in the waiting-for-treatment group, either between the two groups or within the study group. There was no significant difference in the accuracy rate for alcohol-related and non-alcohol-related cues between the two groups or within the study group after rTMS intervention. 3. The total score of MoCA was significantly increased, and the total score of BIS-II was significantly decreased in the study group after rTMS treatment, either between the two groups or within the study group.

**Conclusion:**

The results suggested that high-frequency rTMS could improve the attention bias of alcohol-related cues and impulsivity for patients with AUD.

## Introduction

Alcohol use disorder (AUD) is clinically defined as a loss of control over alcohol intake and risky alcohol intake, maintaining cues despite negative consequences, social impairment, and pharmacological dependence. It is the second most common substance use disorder in the general population after tobacco use disorder (1), with a 12-month and lifetime prevalence in the total population of 13.9 and 29.1%, respectively (2). Craving, defined as a strong and uncontrollable desire to use a substance (3), is one of the fundamental aspects of substance dependency and has been demonstrated to be one of the most critical variables contributing to AUD relapse (4).

In the cognitive theories of alcohol attention bias (AAB) (5), it is hypothesized that when the dopamine system is repeatedly exposed to the rewarding effects of alcohol, it develops to correlate “wanting” with alcohol-related information, resulting in cravings and “loss of control.” Alcohol messages are motivated and given preferred attention automatically and unconsciously, once this relationship is established. Noël et al. (6) suggested that addiction-related behaviors could be gradually controlled by addiction-related information that acquires the property of automatically producing drinking-related behaviors and cravings through Pavlovian and instrumental learning mechanisms (7). At the cognitive processing level, continued drinking leads to implicit “wanting” motivation-related enhancement of associative memory (8), and addiction-related cues are marked as salient cues that capture the addict's attention (9), generating automatic approach tendencies. Cue-induced craving gradually increases in the early stages of abstinence and remains high for a more extended period (10, 11). A quick burst of acute craving enters the consciousness after exposure to the cue, followed by a restart of drinking. In well-controlled laboratory and clinical settings, cue-induced craving could reliably predict relapse across environmental settings and different types of addiction (10, 12). The classic oddball paradigm (13–15) reflects subjects' bias toward alcohol-related cue attention and has important implications for studying alcohol-related cue attention in patients with AUD.

Repetitive transcranial magnetic stimulation (rTMS) has emerged as a promising treatment for substance dependence due to its potential to suppress cravings (16). Studies suggested that excitatory rTMS in the dorsolateral prefrontal cortex (DLPFC) reduced craving in patients with substance dependence (17). However, there are few studies on the effect of rTMS treatment on the spontaneous attentional bias of alcohol cues and impulse processing in patients with AUD. Therefore, this study investigated the effect of rTMS on attention cue reactivity, impulsiveness, and cognitive function in patients with AUD. We hypothesized that consecutive rTMS could improve the attentional bias, impulsiveness, and cognitive function in AUD.

## Objects and Methods

### Objects

A cohort of 90 male subjects with AUD was recruited from the Second Affiliated Hospital of Kunming Medical University and Mental Health Hospital of Yunnan Province. All subjects met the criteria for the Diagnosis and Statistics of Mental Disorder 5^th^ edition (DSM-5) for AUD, with normal vision and hearing or within the normal range after correction and were right-handed. To rule out the influence of acute withdrawal, it was required no alcohol was consumed in the 72 h before the experiment.

The exclusion criteria were (1) Clinical Institute Withdrawal Assessment Alcohol Scale-Revised (CIWA-Ar) (18) score >9 points in acute alcohol withdrawal reaction stage; (2) have experienced a traumatic brain injury or other brain tissue damage; (3) have severe neurological or psychiatric disorders caused by diseases other than chronic alcohol dependence; (4) contraindications to the use of TMS, such as pacemakers, hearing aids, and intracranial metal implants, and a history of epilepsy; (5) diagnosis of other substance use disorders; (6) history of serious physical diseases, including cardiovascular disease and neurological diseases; and (7) have depressed mood and anxiety symptoms rated by Chinese versions of the 9-item Patient Health Questionnaire (PHQ-9) (19) for depressive symptoms (PHQ-9 >5 points), the 7-item Generalized Anxiety Disorder scale (GAD-7) for generalized anxiety symptoms (GAD-7 >5 points) (20).

This study was approved by the Ethics Committee of the Second Affiliated Hospital of Kunming Medical University. All participants provided written informed consent and participated in the study voluntarily. The registration number of this study is NTC 03910686.

### Methods

#### Measures

##### Self-Designed General Information Checklist

The checklist included age, education level, daily alcohol consumption, drinking year, and alcohol consumption variety.

##### Alcohol Dependence Scale

The Alcohol Dependence Scale (ADS) consists of 25 questions, and the scores were recorded as 0, 1, or 2 in the order of each question, and the total score of alcohol dependence (0–47) was obtained by adding up the scores of each question. The score of 0 indicates no alcohol dependence; the score of 1–13 indicates low alcohol dependence; the score of 14–21 indicates moderate alcohol dependence; the score of 22–30 indicates severe alcohol dependence; and the score of 31–47 indicates severe alcohol dependence (21).

##### Montreal Cognitive Assessment

Montreal Cognitive Assessment (MoCA) (22) was used for the screening of cognitive function in patients with AUD. There are six dimensions, namely, visuospatial/executive, naming, attention, language, abstraction, memory, and orientation. The optimal cutoff point of the MoCA to detect cognitive impairment in the general population is a score of <26 (23).

##### Barratt Impulsiveness Scale

**Barratt Impulsiveness Scale** II (BIS-II) is a self-report measure for assessing individual impulsive personality traits (24). The Chinese version of BIS-II has 26 items and shows good reliability and validity (25). Each subscale uses a 4-point scale: “never” is rated as 1, “occasionally” is rated as 2, “often” is rated as 3, and “always” is rated as 4. The higher the score, the stronger the impulsivity.

##### Attention Cue Response Task

**Figure 1:**
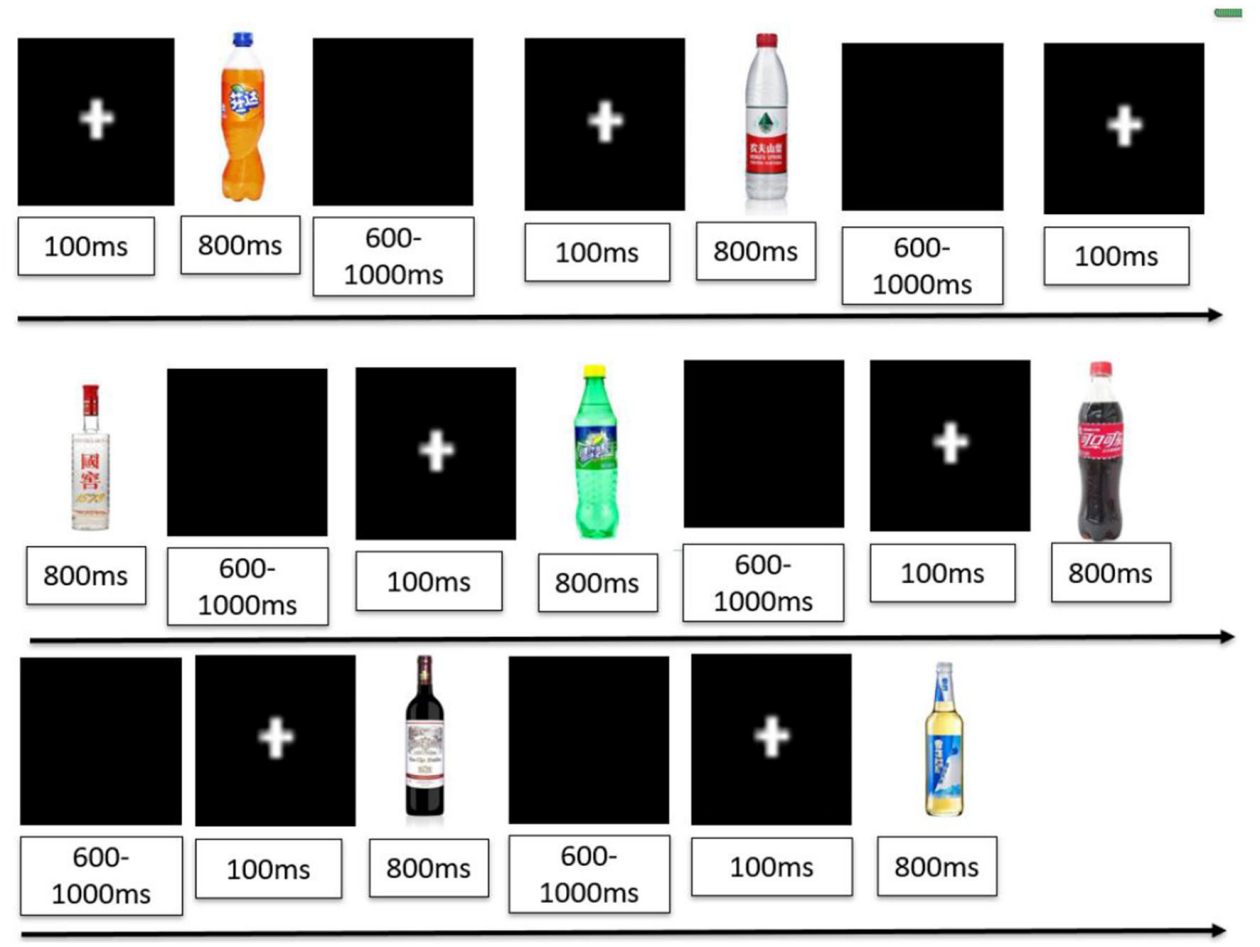
Cue response task-visual oddball paradigm.

As shown in Figure 1, a visual oddball paradigm was used to evaluate the attention cue response. The task and its operation were explained to the subjects before starting the formal experiment. The experiment was not started until the patient passed the exercise unit.

Subjects were presented with the visual oddball paradigm containing repeated high-frequency stimuli (e.g., pictures of a bottle of neutral mineral water) and low-frequency-biased stimuli, including pictures of a bottle of alcohol-related drinks (e.g., beer, liquor, or wine) and non-alcohol-related drinks (e.g., Sprite, Coke, or Fanta). The total number of stimuli was 840. In each block, 75% were high-frequency stimuli (*n* = 120) and 25% were low-frequency stimuli (*n* = 30). Among the low-frequency stimuli, alcohol-related-biased stimuli and non-alcohol-related-biased stimuli, each appeared 15 times. The “+” symbol appeared in the center of the screen for 100 ms before the pictures were presented, attracting the subjects to focus their attention. Each picture was then displayed for 800 ms, with a black screen randomly displayed for 600–1,000 ms between the two pictures. Subjects were given at least 1,400 ms from the onset of the stimulus to respond and were asked to indicate the onset of any low-frequency stimulus by tapping the space bar with their right finger as quickly and accurately as possible. The reaction time and accuracy rate of subjects were recorded. The attention cue response task was reevaluated after the 14 rTMS interventions.

### Procedure

**Figure 2:**
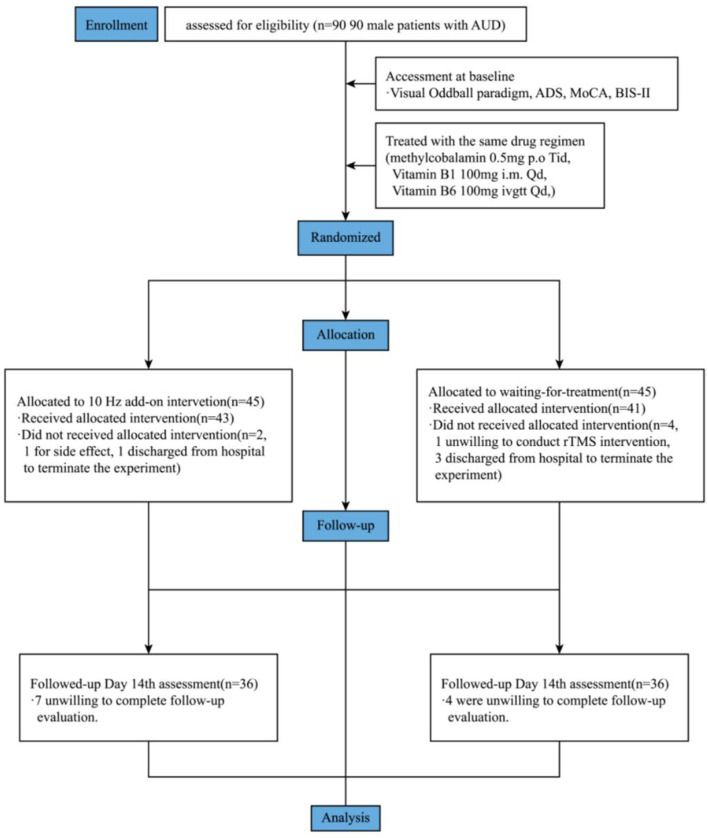
Flowchart of study. ADS, Alcohol dependence scale. MoCA, Montreal Cognitive Assessment; BIS-II, Barratt impulsiveness scale.

As shown in Figure 2, after CIWA-Ar score <9 points, demonstrating that the subjects were not in an acute alcohol withdrawal stage, a total of 90 male patients with AUD were treated with the same drug regimen: methylcobalamin 0.5 mg *p.o* Tid, vitamin B_1_ 100 mg *i.m* Qd, and vitamin B_6_ 100 mg *ivgtt* Qd.

A random number table was used to divide the subjects into 45 in the study group (i.e., actual stimulation group) and 45 in the waiting-for-treatment group (i.e., pseudo-stimulation group). Except for the operator, the subjects were blind to true or false stimulation in the rTMS trial. The wait-for-treatment group would continue to receive actual stimulation for 2 weeks after the 2-week study is completed, but the data would not include in this study. All subjects were evaluated by using the visual oddball paradigm, MoCA, and BIS-II at baseline and after rTMS.

There were 18 dropouts during the study. Reasons for dropping out were as follows. There were 11 patients unwilling to complete the follow-up evaluation, 2 patients unwilling to conduct rTMS intervention, 1 patient discontinued rTMS due to side effects, and 4 patients discharged from the hospital to terminate the experiment. Finally, 36 subjects in the study group and 36 subjects in the wait-for-treatment group completed the experiment.

### rTMS Intervention

**Figure 3:**
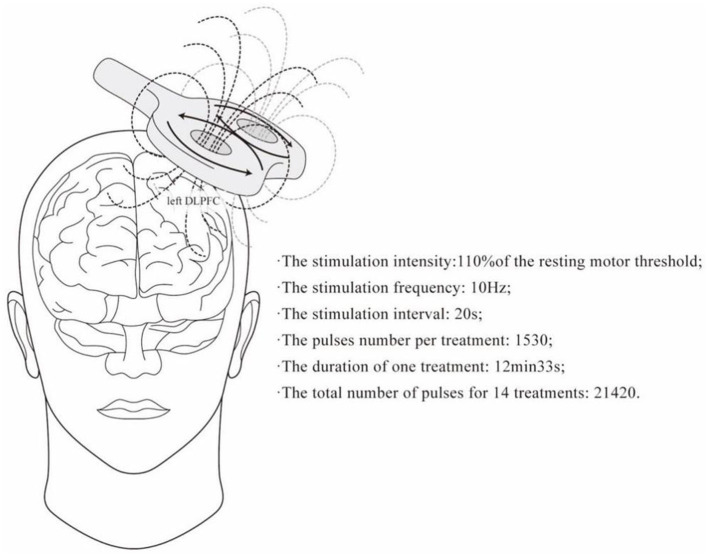
Protocol and parameters of rTMS intervention.

Repetitive transcranial magnetic stimulation was administered using a CCY-1 TMS stimulator (Eride Inc, Wuhan, China) equipped with 8 coils. Patients in the study group received rTMS treatment for 14 consecutive days once a day. The stimulation site was selected as left DLPFC (17), and the international 10–20 EEG system (F3) was used for localization. The motion thresholds of individual magnetic stimuli were measured. Treatment parameters were set as shown in Figure 3: the stimulation intensity: 110% of the resting motor threshold; the stimulation frequency: 10 Hz; the stimulation interval: 20 s; the number of pulses per treatment: 1,530; the duration of one treatment: 12 min and 33 s; and the total number of pulses for 14 treatments: 21,420. For sham stimulation, the stimulation coil was tilted at a 90°angle to the scalp, and the coil was spaced approximately 3 cm apart from the scalp to reduce the effect of the magnetic field on the brain (17). After rTMS treatment, patients were invited to report adverse reactions.

### Statistical Processing

Statistical analyses were performed using SPSS 21.0 (Statistical Package for Social Sciences, IBM, Armonk, NY). Continuous data were presented as mean ± standard deviation (SD) or median [interquartile range]. Categorical data were presented as absolute numbers and percentages. The demographic and clinical characteristics of the two groups were compared by using the one-way ANOVA, Kruskal-Wallis H-test, or Fisher's exact test. The pre-post results of attention cue response after rTMS were compared using a paired-sample *t*-test. The correlation between BIS-II scores and results of the oddball paradigm, MoCA after rTMS in the study group were analyzed using the Spearman correlation analysis. We used a two-sided α of 0.05 for statistical significance.

## Results

### Demographic and Clinical Characteristics Between the Two Groups

As shown in Table 1, for the mean age, education, type of alcohol consumption, daily alcohol consumption, drinking year, and scores of ADS, PHQ-9, GAD-7, MoCA, and BIS-II, there were no significant differences between the study group and the waiting-for-treatment group at baseline (*P* > 0.05). After rTMS, for the scores of PHQ-9 and GAD-7, there were no significant differences between the two groups.

**Table 1:** Comparison of basic demographic information between the study group and the waiting-for- treatment group [x ± s/*n* (%)].

	**Study group (*n* = 45)**	**Waiting-for-treatment group (*n* = 45)**	***t / χ^2^*** **/ z**	***P***
Age	37.74 ± 6.42	38.11 ± 5.33	−0.273	0.785
Drinking year	17.39 ± 6.51	18.43 ± 6.01	−0.704	0.484
Educational level				
Primary	7 (22.22)	2 (5.56)	−0.476	0.634
Junior secondary school	7 (19.44)	8 (22.22)		
High school	12 (33.33)	19 (52.78)		
University	10 (25)	7 (19.44)		
Type of alcohol consumption			−1.381	0.167
White Wine	32 (88.89)	35 (97.22)		
Beer	4 (11.11)	1 (2.78)		
Daily alcohol consumption(g)	141.43 ± 29.46	132.54 ± 39.89	1.076	0.286
ADS	30.26 ± 5.16	31.78 ± 4.57	−1.323	0.190
GAD-7	3.07 ± 0.88	3.15 ± 0.76	−0.413	0.681
PHQ-9	2.89 ± 0.82	3.11 ± 0.71	−1.175	0.244
MoCA	22.91 ± 1.98	23.04 ± 1.74	−0.296	0.768
BIS-II	62.30 ± 9.92	66.56 ± 11.26	−1.703	0.093

### Pre-post Comparison of Attention cue Response After rTMS

Before rTMS intervention, there were no significant differences in response time and accuracy rate between the two groups, neither in alcohol-related cues nor in non-alcohol-related cues.

As shown in Table 2, after 14 rTMS interventions, both for alcohol-related cues and non-alcohol-related cues, the response times were significantly shorter in the study group compared with those in the waiting-for-treatment group. Within the study group, the response times for two kinds of cues after rTMS were shorter than baseline.

**Table 2:** Pre-post comparison of response time (ms) and accuracy rate (%) after rTMS in the oddball paradigm.

	**Study group (*N* = 36)**	**Waiting-for-treatment (*N* = 36)**	**t**	***P***
**Response time of alcohol–related cues**
Pre- rTMS	530.44 ± 71.31	531.50 ± 80.58	−0.059	0.953
Post- rTMS	499.31 ± 62.97	530.69 ± 58.15	−2.197	0.031^*^
t	2.306	0.051		
*P*	0.027^*^	0.959		
**Response time of non-alcohol-related cues**
Pre- rTMS	531.64 ± 70.31	530.75 ± 71.87	0.053	0.958
Post- rTMS	493.14 ± 64.79	526.47 ± 60.77	−2.251	0.027 ^*^
t	2.504	0.235		
*P*	0.017 ^*^	0.815		
**Accuracy rate of alcohol-related cues**
Pre- rTMS	0.97 ± 0.05	0.98 ± 0.04	−0.583	0.562
Post- rTMS	0.98 ± 0.05	0.98 ± 0.05	−0.189	0.851
t	0.594	0.271		
*P*	0.557	0.788		
**Accuracy rate of non-alcohol-related cues**
Pre- rTMS	0.99 ± 0.06	0.97 ± 0.04	0.93	0.355
Post- rTMS	0.99 ± 0.04	0.98 ± 0.05	0.69	0.492
t	0.368	0.713		
*P*	0.715	0.480		

* *P < 0.05*.

There was no significant difference in accuracy rates for both alcohol-related and non-alcohol-related cues between the two groups after rTMS intervention. Within the study group or waiting-for-treatment group itself, there was also no significant difference in accuracy rates for two kinds of cues after rTMS.

### Pre-post Comparison of BIS-II and MoCA After rTMS

As shown in Table 3, the total score of MoCA was significantly increased, and the total score of BIS-II was significantly decreased in the study group after rTMS treatment, either between the two groups or within the study group itself. For the waiting-for-treatment group, there was no significant difference in the total scores of MoCA and BIS-II after sham rTMS.

**Table 3:** Pre-post comparison of BIS-II, MoCA after rTMS.

	**Study group (*N* = 36)**	**Waiting–for–treatment (*N* = 36)**	**t**	***P***
**Total score of BIS-II**
Pre- rTMS	65.86 ± 10.89	66.75 ± 10.23	−1.877	0.148
Post- rTMS	55.10 ± 7.75	65.41 ± 10.49	−19.197	<0.001
t	21.54	0.981		
*P*	<0.001	0.959		
**Total score of MoCA**
Pre- rTMS	20.04 ± 3.83	20.86 ± 4.07	0.071	0.548
Post- rTMS	23.43 ± 4.09	21.03 ± 4.18	3.253	0.029*
t	−10.681	0.935		
*P*	<0.001	0.725		

### The Correlation Between BIS-II Scores and Results of the Oddball Paradigm, MoCA After rTMS in the Study Group

There was no significant correlation between the BIS-II score and MoCA scores after rTMS. However, as shown in Table 4, there was a significantly negative correlation between change in BIS-II and change in response time of alcohol-related cues or change in response time of non-alcohol-related cues after rTMS.

**Table 4:** The correlation between BIC-II scores, results of the Oddball paradigm, and MoCA after rTMS in the study group (*n* = 36).

	**Change of BIS-II (r, *P*)**	**Change of MoCA (r, *P*)**
Change of response time of alcohol-related cues	−0.419, 0.011	0.515, 0.001
Change of response time of non-alcohol-related cues	−0.477, 0.003	0.639, <0.001
Change of accuracy rate of alcohol-related cues	−0.207, 0.226	0.113, 0.511
Change of accuracy rate of non-alcohol-related cues	−0.269, 0.113	0.067, 0.700

There was a significantly positive correlation between the change in MoCA and response time of alcohol-related cues or the change in response time of non-alcohol-related cues after rTMS. Spearman's correlation analysis showed further a significant positive association between the change in attention score in MoCA and the change in response time of alcohol-related cues (F = 7.838, *P* = 0.001), or the change in response time of non-alcohol-related cues (F = 7.016, *P* = 0.009) after rTMS. Another significant positive association between the change in Memory and Orientation score in MoCA and the change in response time of alcohol-related cues (F = 8.252, *P* < 0.001) or the change in response time of non-alcohol-related cues (F = 9.067, *P* < 0.001) was found after rTMS.

There were no significant correlations found between the score of BIS-II and MoCA and the change in the accuracy rate of alcohol-related cues or non-alcohol-related cues.

### Side Effects During rTMS Stimulation

As shown in Table 5, there were some side effects during rTMS stimulation, especially in the actual rTMS group. These side effects included headache, tinnitus, dizziness, and eye discomfort, most of which were mild degrees and relieved after stopping rTMS stimulation. Only one patient discontinued rTMS due to a headache.

**Table 5:** Side effects during rTMS stimulation.

**Side effects**	**Study group (*n* = 36)**	**Waiting-for-treatment group (*n* = 36)**
Headache	2	0
Tinnitus	3	1
Dizzy	2	0
Eye discomfort	1	0

## Discussion

The evidence suggested that the orbitofrontal cortex of patients with AUD could be strongly activated by alcohol-related cues (26) and that activation of this region was associated with craving and relapse into drinking (27). Patients with AUD with impaired executive function were more inclined to drinking due to impulse processing, thus creating a vicious circle (28).

In this study, there were no statistical differences in the mean age, education, daily drinking, year of drinking, level of alcohol dependence, cognitive function, impulsivity, response times, and accuracy rate for the two types of cue stimuli (e.g., alcohol-related cue and non-alcohol-related cue) between the two groups of patients at the baseline, suggesting that the subjects in two groups were at the same baseline level in terms of demographic profile, drinking and severity, and cognitive level.

After high-frequency rTMS treatment at 10 Hz for a continued 14 days, the reaction time of an alcohol-related cue and non-alcohol-related cue in the study group were both shorter when compared with the baseline or the waiting-for-treatment group. Psychomotor vigilance and sustained and selective attention are reflected by shorter reaction times (29). Recently, in a systemic review, it was found that rTMS could influence the attentional networks in alcohol-dependent and other addicted patients (30). These results indicated that high-frequency rTMS acting on the left DLPFC could help to improve attentional drift and bias in patients with AUD after acute detoxification. The total score of MoCA was significantly increased, and the total score of BIS-II was significantly decreased in the study group, suggesting that rTMS improved cognitive function and decreased impulsivity for patients with AUD. Spearman's correlation analysis further demonstrated that the level of BIS-II negatively correlated with the improvement of response time in two kinds of attention cue response, and the improvement of MoCA and reaction time was mutually reinforcing, suggesting that decreased impulsivity and improved cognitive function, especially the improvement of attention, memory, and orientation, could also be helpful for the improvement of attentional bias after rTMS. These results were consistent with previous studies that rTMS treatment could improve alcohol craving, cognitive function, and heavy drinking (31–33) and affect the dorsal anterior cingulate cortex (34). Alcohol-attentional bias and impulsive decision-making are vulnerability markers for maintaining addiction-like behaviors (29). A potential candidate mechanism of rTMS acting on the left DLPFC could be that rTMS modulates the attention bias to alcohol-related cues and impulsivity.

However, after 14 days of treatment, there was no significant difference in the accuracy rate for alcohol-related cues and non-alcohol-related cues between the two groups after rTMS intervention. Although high-frequency rTMS treatment significantly improved the reaction time, patients with AUD generally had deficits of inhibition ability resistance to interference ability reflected by lower commission error number and accuracy rates (35). The damage by alcohol to cognitive function would persist longer. Previous studies demonstrated that the cognitive dysfunction in AUD did not completely recover after prolonged abstinence and remained lower when compared with controls (36). In this study, 14 days of rTMS intervention was only short-term treatment. It might be that a more extended treatment period or more comprehensive treatment approaches are needed to improve cognitive deficits further.

In this study, some side effects mainly happened in the actual rTMS group, most of which were to mild degrees and relieved after stopping rTMS stimulation. The safety of rTMS continued to be supported by meta-analyses, or evidence-based guidelines (37, 38), demonstrating that rTMS is a promising non-invasive treatment for various neuropsychiatric conditions, including AUD.

There were several limitations of this study. First, the standard 10–20 EEG partitions were utilized for brain area localization during the rTMS intervention in this study, and the accuracy might be enhanced by utilizing more EEG recorder leads. Second, due to 18 dropouts, the number of enrolled subjects decreased. Therefore, the results needed more replication in a larger sample. Although the comparison between the two groups or pre-post rTMS was made, the healthy control group is needed in future research, especially for the results of the oddball paradigm at baseline. We used the classic oddball task for the oddball paradigm, which required subjects to respond to the target stimuli (low-frequency stimuli) but not to the standard stimuli (high-frequency stimuli), so we could not record the reaction time and accuracy rate of unattended stimuli. In the later research, a two-choice oddball task (39) should be used to reflect the ability of behavioral inhibitory control, and both reaction time and accuracy rate in standard stimuli could be recorded. In addition, this study was conducted using a single-blind method, and the results are yet to be confirmed using a double-blind methodology. Furthermore, clinical evaluations such as MoCA and BIS-II were assessed using self-rating scales by patients themselves. All these evaluations, including the visual oddball paradigm, were repeatedly assessed after 2 weeks; thus, some subjective bias or subjective bias recall error might have occurred. In addition, more extended follow-up observation is needed to demonstrate how long the change of clinical evaluations would be persistent after discontinuation of rTMS. Finally, combined application of event-related potentials and eye-tracking could be used to evaluate how brain functions are enhanced in the future.

## Conclusion

This study revealed that high-frequency rTMS treatment at 10 Hz for continuous 14 days could improve the attention bias of alcohol-related cues and impulsivity in patients with AUD.

## Data Availability Statement

The original contributions presented in the study are included in the article/supplementary material, further inquiries can be directed to the corresponding authors.

## Ethics Statement

The studies involving human participants were reviewed and approved by the Ethics Committee of the Second Affiliated Hospital of Kunming Medical University. The patients/participants provided their written informed consent to participate in this study.

## Author Contributions

ZF performed data collection, data analysis, data interpretation, and manuscript writing. QW and LW conducted data collection, statistical analysis and data interpretation, and manuscript preparation. QW wrote the manuscript of the Chinese version. TZ, JYu, and XW collected the data. JYa and CK were responsible for project design, data analysis, and manuscript writing and modification. All authors contributed to the article and approved the submitted version.

## Funding

This study was supported by the National Key R&D Program of China [Grant Number: 2018YFC 1314400, and 2018YFC 1314405]. The grant had no further role in the study design, the collection, analysis, and interpretation of data, the report's writing, or the decision to submit the article for publication.

## Conflict of Interest

The authors declare that the research was conducted in the absence of any commercial or financial relationships that could be construed as a potential conflict of interest.

## Publisher's Note

All claims expressed in this article are solely those of the authors and do not necessarily represent those of their affiliated organizations, or those of the publisher, the editors and the reviewers. Any product that may be evaluated in this article, or claim that may be made by its manufacturer, is not guaranteed or endorsed by the publisher.
